# 4-Benzyl-4-ethyl­morpholin-1-ium hexa­fluoro­phosphate

**DOI:** 10.1107/S1600536812001006

**Published:** 2012-02-24

**Authors:** Fang Yang, Hongjun Zang, Bowen Cheng, Xianlin Xu, Yuanlin Ren

**Affiliations:** a Department of Enviromental and Chemistry Engineering, Tianjin Polytechnic University, State Key Laboratory of Hollow Fiber Membrane Materials and Processes, Tianjin 300160, People’s Republic of China

## Abstract

The asymmetric unit of the title compound, C_13_H_20_NO^+^·PF_6_^−^, contains two cations, one complete anion and two half hexa­fluoro­phosphate anions having crystallographically imposed twofold rotation symmetry. In the cations, the morpholine rings are in a chair conformation. In the crystal, ions are linked by weak C—H⋯F hydrogen bonds into a three-dimensional network.

## Related literature

For background to the properties and applications of quaternary ammonium-based compounds as room temperature ionic liquids (RTILs), see: Abedin *et al.* (2004 ▶, 2005 ▶); Kim *et al.* (2006 ▶). For ring puckering parameters, see: Cremer & Pople (1975 ▶) For bond-length data, see: Allen *et al.* (1987 ▶).
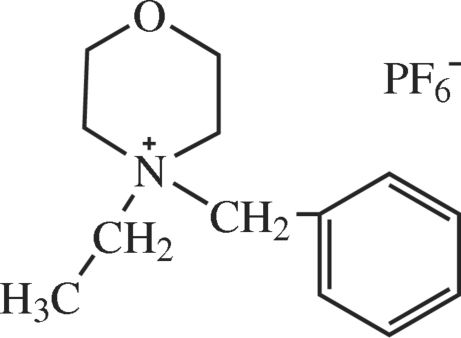


## Experimental

### 

#### Crystal data

C_13_H_20_NO^+^·PF_6_^−^
*M* *_r_* = 351.27
Orthorhombic, 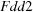

*a* = 26.054 (3) Å
*b* = 28.528 (3) Å
*c* = 16.2950 (15) Å
*V* = 12111 (2) Å^3^
*Z* = 32
Mo *K*α radiation
μ = 0.25 mm^−1^
*T* = 113 K
0.22 × 0.20 × 0.18 mm

#### Data collection

Rigaku Saturn diffractometer
Absorption correction: multi-scan (*CrystalClear*; Rigaku/MSC, 2005 ▶) *T*_min_ = 0.948, *T*_max_ = 0.957
39690 measured reflections
8072 independent reflections
7799 reflections with *I* > 2σ(*I*)
*R*_int_ = 0.042

#### Refinement

*R*[*F* ^2^ > 2σ(*F* ^2^)] = 0.036
*wR*(*F* ^2^) = 0.078
*S* = 1.11
8072 reflections
402 parameters
1 restraint
H-atom parameters constrained
Δρ_max_ = 0.31 e Å^−3^
Δρ_min_ = −0.24 e Å^−3^
Absolute structure: Flack (1983 ▶), 3877 Friedel pairs
Flack parameter: −0.06 (5)

### 

Data collection: *CrystalClear* (Rigaku/MSC, 2005 ▶); cell refinement: *CrystalClear*; data reduction: *CrystalClear*; program(s) used to solve structure: *SHELXS97* (Sheldrick, 2008 ▶); program(s) used to refine structure: *SHELXL97* (Sheldrick, 2008 ▶); molecular graphics: *SHELXTL* (Sheldrick, 2008 ▶); software used to prepare material for publication: *CrystalStructure* (Rigaku, 2007 ▶).

## Supplementary Material

Crystal structure: contains datablock(s) I, global. DOI: 10.1107/S1600536812001006/rz2692sup1.cif

Structure factors: contains datablock(s) I. DOI: 10.1107/S1600536812001006/rz2692Isup2.hkl

Additional supplementary materials:  crystallographic information; 3D view; checkCIF report

## Figures and Tables

**Table 1:** Hydrogen-bond geometry (Å, °)

*D*—H⋯*A*	*D*—H	H⋯*A*	*D*⋯*A*	*D*—H⋯*A*
C1—H1*A*⋯F4	0.99	2.41	3.211 (2)	138
C3—H3*B*⋯F2^i^	0.99	2.47	3.389 (2)	155
C5—H5*B*⋯F11^ii^	0.99	2.48	3.365 (2)	150
C10—H10⋯F10^iii^	0.95	2.50	3.288 (2)	141
C17—H17*A*⋯F8^iv^	0.99	2.37	3.329 (2)	162
C17—H17*B*⋯F1	0.99	2.46	3.357 (2)	150
C25—H25*B*⋯F1	0.99	2.53	3.388 (2)	145

Symmetry codes: (i) 
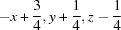
; (ii) 
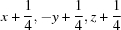
; (iii) 
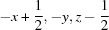
; (iv) 
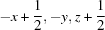
.

**Fig. 1.**
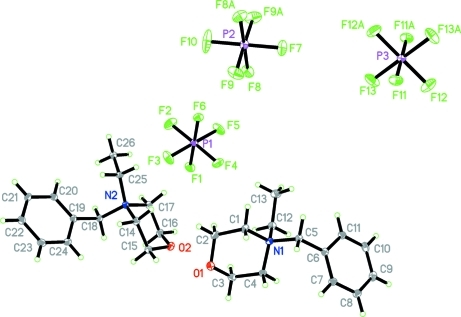
The asymmetric unit of the title compound showing displacement ellipsoids drawn at the 30% probability level. Symmetry code: (A) -x, -y, z.

**Fig. 2.**
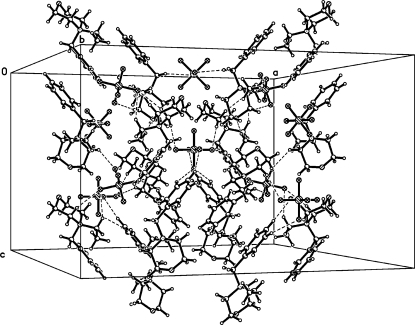
Crystal packing of the title compound. Interionic hydrogen bonds are shown as dashed lines.

## supplementary crystallographic information

### Comment

The seemingly ever growing interest on room temperature ionic liquids (RTILs) has turned the spotlight, along with many others, to quaternary ammonium-based compounds. The excellent conductivity, broad electrochemical window, thermal stability, and low volatility of ILs have made them promising media for electrochemical processes (Abedin *et al.*, 2004; Abedin *et al.*, 2005). RTILs based on the morpholinium cation are favored because of their low cost, easy synthesis, and electrochemical stability (Kim *et al.*, 2006). We report here a new example structure of this class of compounds.

The asymmetric unit of the title compound (Fig. 1) consists of two cations, one PF_6_^-^ anion and two half of PF_6_^-^ anion having crystallographically imposed twofold rotation symmetry. In the cations, the morpholine rings adopt a chair conformation, with puckering parameters *Q*, θ and φ (Cremer & Pople, 1975) of 0.5757 (17) Å, 4.07 (14)°, 169 (2)° and 0.5607 (15) Å, 4.15 (14)°, 177 (3)° for N1/C1/C2/O1/C3/C4 and N2/C14/C15/O2/C16/C17, respectively. All bond distances and angles in the cation are normal within experimental error (Allen *et al.*, 1987). In the crystal packing, cations and anions are involved in weak C—H···F hydrogen bonds (Table 1) linking ions into a three-dimensional network (Fig. 2).

### Experimental

*N*-Benzyl-*N*-ethylmorpholinium hexafluorophosphate was synthesized by dissolving the *N*-benzyl-*N*-ethylmorpholinium chloride (12.1 g, 0.05 mol) in a minimum volume of deionized water and adding the stoichiomeric amount (1:1) of 60% HPF_6_ solution. The mixture was stirred for about 5 min at 0°C causing the precipitation of a quaternary ammonium hexafluorophosphate from the solution. The raw product was filtered and washed with water until the solution was neutral. The product was recrystallized from the methanol/ethyl acetate solvent (1:1 *v*/*v*) and dried *in vacuo*.

### Refinement

All H atoms were positioned geometrically and included in the refinement in the riding model approximation, with C–H = 0.95–0.99 Å and *U*_iso_ (H) = 1.2 *U*_eq_(C) or 1.5*U*_eq_(C) for methyl H atoms.

### Crystal data

**Table:**

C_13_H_20_NO^+^·PF_6_^−^	*F*(000) = 5824
*M**_r_* = 351.27	*D*_x_ = 1.541 Mg m^−^^3^
Orthorhombic, *F**d**d*2	Mo *K*α radiation, λ = 0.71070 Å
Hall symbol: F 2 -2d	Cell parameters from 11300 reflections
*a* = 26.054 (3) Å	θ = 2.1–29.1°
*b* = 28.528 (3) Å	µ = 0.25 mm^−^^1^
*c* = 16.2950 (15) Å	*T* = 113 K
*V* = 12111 (2) Å^3^	Block, colourless
*Z* = 32	0.22 × 0.20 × 0.18 mm

### Data collection

**Table:**

Rigaku Saturn diffractometer	8072 independent reflections
Radiation source: rotating anode	7799 reflections with *I* > 2σ(*I*)
Confocal monochromator	*R*_int_ = 0.042
Detector resolution: 14.63 pixels mm^-1^	θ_max_ = 29.1°, θ_min_ = 2.1°
ω and φ scans	*h* = −35→35
Absorption correction: multi-scan (*CrystalClear*; Rigaku/MSC, 2005)	*k* = −38→38
*T*_min_ = 0.948, *T*_max_ = 0.957	*l* = −22→22
39690 measured reflections	

### Refinement

**Table:**

Refinement on *F*^2^	Hydrogen site location: inferred from neighbouring sites
Least-squares matrix: full	H-atom parameters constrained
*R*[*F*^2^ > 2σ(*F*^2^)] = 0.036	*w* = 1/[σ^2^(*F*_o_^2^) + (0.0336*P*)^2^ + 3.6844*P*] where *P* = (*F*_o_^2^ + 2*F*_c_^2^)/3
*wR*(*F*^2^) = 0.078	(Δ/σ)_max_ = 0.001
*S* = 1.11	Δρ_max_ = 0.31 e Å^−^^3^
8072 reflections	Δρ_min_ = −0.24 e Å^−^^3^
402 parameters	Extinction correction: *SHELXL97* (Sheldrick, 2008), Fc^*^=kFc[1+0.001xFc^2^λ^3^/sin(2θ)]^-1/4^
1 restraint	Extinction coefficient: 0.00044 (2)
Primary atom site location: structure-invariant direct methods	Absolute structure: Flack (1983), 3877 Friedel pairs
Secondary atom site location: difference Fourier map	Flack parameter: −0.06 (5)

### Special details

**Table:**

Geometry. All e.s.d.'s (except the e.s.d. in the dihedral angle between two l.s. planes) are estimated using the full covariance matrix. The cell e.s.d.'s are taken into account individually in the estimation of e.s.d.'s in distances, angles and torsion angles; correlations between e.s.d.'s in cell parameters are only used when they are defined by crystal symmetry. An approximate (isotropic) treatment of cell e.s.d.'s is used for estimating e.s.d.'s involving l.s. planes.
Refinement. Refinement of *F*^2^ against ALL reflections. The weighted *R*-factor *wR* and goodness of fit *S* are based on *F*^2^, conventional *R*-factors *R* are based on *F*, with *F* set to zero for negative *F*^2^. The threshold expression of *F*^2^ > σ(*F*^2^) is used only for calculating *R*-factors(gt) *etc*. and is not relevant to the choice of reflections for refinement. *R*-factors based on *F*^2^ are statistically about twice as large as those based on *F*, and *R*- factors based on ALL data will be even larger.

### Fractional atomic coordinates and isotropic or equivalent isotropic displacement parameters (Å^2^)

**Table:**

	*x*	*y*	*z*	*U*_iso_*/*U*_eq_	
P1	0.243907 (17)	0.017672 (16)	0.61924 (3)	0.02020 (9)	
P2	0.0000	0.0000	0.43883 (4)	0.02273 (14)	
P3	0.0000	0.0000	0.04698 (4)	0.02275 (14)	
F1	0.30299 (4)	0.02779 (4)	0.64111 (7)	0.0343 (3)	
F2	0.23883 (5)	−0.01213 (5)	0.70168 (8)	0.0478 (4)	
F3	0.22767 (5)	0.06362 (5)	0.66893 (8)	0.0449 (3)	
F4	0.24903 (4)	0.04840 (4)	0.53785 (8)	0.0396 (3)	
F5	0.26089 (5)	−0.02761 (4)	0.56973 (9)	0.0432 (3)	
F6	0.18516 (4)	0.00756 (4)	0.59767 (7)	0.0275 (2)	
F7	0.0000	0.0000	0.34159 (10)	0.0413 (4)	
F8	0.06135 (4)	0.00085 (4)	0.43940 (9)	0.0401 (3)	
F9	−0.00157 (5)	0.05575 (4)	0.43761 (11)	0.0507 (4)	
F10	0.0000	0.0000	0.53569 (11)	0.0735 (8)	
F11	−0.01702 (5)	0.05392 (4)	0.04710 (8)	0.0372 (3)	
F12	0.04163 (6)	0.01103 (5)	−0.02151 (9)	0.0525 (4)	
F13	0.04128 (6)	0.01141 (5)	0.11579 (9)	0.0555 (4)	
N1	0.35571 (5)	0.11940 (5)	0.37982 (8)	0.0178 (3)	
N2	0.35425 (5)	0.08706 (5)	0.82302 (8)	0.0171 (3)	
O1	0.36473 (5)	0.17498 (5)	0.52911 (8)	0.0294 (3)	
O2	0.41528 (5)	0.12098 (4)	0.68472 (8)	0.0272 (3)	
C1	0.31222 (6)	0.12724 (6)	0.43997 (11)	0.0220 (3)	
H1A	0.2893	0.0996	0.4395	0.026*	
H1B	0.2919	0.1548	0.4223	0.026*	
C2	0.33174 (7)	0.13527 (7)	0.52658 (11)	0.0266 (4)	
H2A	0.3023	0.1403	0.5640	0.032*	
H2B	0.3506	0.1072	0.5455	0.032*	
C3	0.40842 (7)	0.16670 (7)	0.47901 (11)	0.0281 (4)	
H3A	0.4267	0.1385	0.4989	0.034*	
H3B	0.4322	0.1937	0.4831	0.034*	
C4	0.39294 (6)	0.15971 (6)	0.39020 (11)	0.0211 (3)	
H4A	0.3769	0.1888	0.3694	0.025*	
H4B	0.4240	0.1536	0.3569	0.025*	
C5	0.33253 (6)	0.12094 (6)	0.29343 (10)	0.0197 (3)	
H5A	0.3072	0.0952	0.2883	0.024*	
H5B	0.3138	0.1509	0.2868	0.024*	
C6	0.37087 (6)	0.11662 (6)	0.22510 (10)	0.0189 (3)	
C7	0.39250 (7)	0.15721 (6)	0.19202 (11)	0.0229 (3)	
H7	0.3839	0.1870	0.2143	0.027*	
C8	0.42658 (7)	0.15427 (6)	0.12656 (11)	0.0254 (4)	
H8	0.4408	0.1820	0.1038	0.030*	
C9	0.43974 (7)	0.11101 (7)	0.09459 (11)	0.0262 (4)	
H9	0.4636	0.1090	0.0507	0.031*	
C10	0.41805 (7)	0.07058 (6)	0.12674 (11)	0.0265 (4)	
H10	0.4269	0.0409	0.1046	0.032*	
C11	0.38354 (7)	0.07350 (6)	0.19097 (11)	0.0223 (3)	
H11	0.3683	0.0457	0.2120	0.027*	
C12	0.38380 (7)	0.07356 (6)	0.39370 (11)	0.0231 (4)	
H12A	0.4018	0.0752	0.4471	0.028*	
H12B	0.4102	0.0700	0.3505	0.028*	
C13	0.34997 (8)	0.03033 (6)	0.39328 (13)	0.0326 (4)	
H13A	0.3293	0.0296	0.4435	0.049*	
H13B	0.3715	0.0022	0.3907	0.049*	
H13C	0.3272	0.0312	0.3454	0.049*	
C14	0.33713 (7)	0.12568 (6)	0.76530 (10)	0.0206 (3)	
H14A	0.3162	0.1118	0.7208	0.025*	
H14B	0.3152	0.1480	0.7958	0.025*	
C15	0.38179 (7)	0.15194 (6)	0.72795 (11)	0.0242 (4)	
H15A	0.3686	0.1761	0.6897	0.029*	
H15B	0.4012	0.1680	0.7719	0.029*	
C16	0.43546 (7)	0.08706 (6)	0.73984 (11)	0.0248 (4)	
H16A	0.4549	0.1032	0.7838	0.030*	
H16B	0.4596	0.0664	0.7100	0.030*	
C17	0.39362 (6)	0.05763 (6)	0.77790 (11)	0.0198 (3)	
H17A	0.4092	0.0351	0.8167	0.024*	
H17B	0.3762	0.0395	0.7343	0.024*	
C18	0.37772 (6)	0.10664 (6)	0.90207 (10)	0.0182 (3)	
H18A	0.3909	0.0802	0.9353	0.022*	
H18B	0.4073	0.1267	0.8875	0.022*	
C19	0.34089 (6)	0.13489 (6)	0.95395 (10)	0.0187 (3)	
C20	0.31333 (6)	0.11352 (6)	1.01675 (10)	0.0196 (3)	
H20	0.3166	0.0807	1.0255	0.024*	
C21	0.28113 (7)	0.13974 (6)	1.06672 (10)	0.0223 (3)	
H21	0.2626	0.1249	1.1096	0.027*	
C22	0.27592 (7)	0.18765 (6)	1.05420 (11)	0.0246 (4)	
H22	0.2536	0.2056	1.0880	0.030*	
C23	0.30346 (7)	0.20911 (6)	0.99205 (12)	0.0272 (4)	
H23	0.3000	0.2419	0.9832	0.033*	
C24	0.33603 (7)	0.18308 (6)	0.94274 (11)	0.0229 (3)	
H24	0.3552	0.1982	0.9009	0.028*	
C25	0.30729 (6)	0.05742 (6)	0.84234 (11)	0.0207 (3)	
H25A	0.2831	0.0766	0.8750	0.025*	
H25B	0.2900	0.0494	0.7901	0.025*	
C26	0.31801 (7)	0.01251 (6)	0.88864 (11)	0.0250 (4)	
H26A	0.3397	−0.0080	0.8551	0.038*	
H26B	0.2855	−0.0034	0.9006	0.038*	
H26C	0.3357	0.0198	0.9402	0.038*	

### Atomic displacement parameters (Å^2^)

**Table:**

	*U*^11^	*U*^22^	*U*^33^	*U*^12^	*U*^13^	*U*^23^
P1	0.0186 (2)	0.0212 (2)	0.0208 (2)	−0.00042 (16)	−0.00207 (16)	0.00038 (16)
P2	0.0198 (3)	0.0267 (3)	0.0217 (3)	0.0065 (2)	0.000	0.000
P3	0.0247 (3)	0.0216 (3)	0.0219 (3)	−0.0064 (2)	0.000	0.000
F1	0.0223 (6)	0.0433 (7)	0.0375 (6)	−0.0046 (5)	−0.0076 (5)	−0.0078 (5)
F2	0.0369 (7)	0.0659 (9)	0.0406 (8)	−0.0026 (6)	−0.0068 (6)	0.0292 (7)
F3	0.0397 (7)	0.0450 (7)	0.0500 (8)	0.0113 (6)	−0.0102 (6)	−0.0243 (6)
F4	0.0343 (7)	0.0502 (8)	0.0343 (6)	−0.0143 (5)	−0.0025 (5)	0.0163 (6)
F5	0.0315 (7)	0.0360 (7)	0.0622 (9)	0.0067 (5)	−0.0092 (6)	−0.0241 (6)
F6	0.0186 (5)	0.0324 (6)	0.0316 (6)	−0.0028 (4)	−0.0041 (4)	0.0049 (5)
F7	0.0446 (11)	0.0559 (12)	0.0235 (8)	0.0184 (8)	0.000	0.000
F8	0.0218 (6)	0.0471 (7)	0.0514 (8)	0.0068 (5)	−0.0020 (6)	−0.0154 (6)
F9	0.0415 (8)	0.0288 (6)	0.0818 (10)	0.0086 (6)	−0.0038 (7)	−0.0173 (7)
F10	0.0552 (13)	0.144 (2)	0.0211 (9)	0.0621 (14)	0.000	0.000
F11	0.0377 (7)	0.0230 (6)	0.0508 (7)	−0.0025 (5)	−0.0003 (6)	0.0017 (5)
F12	0.0502 (9)	0.0517 (8)	0.0555 (9)	0.0037 (6)	0.0270 (7)	0.0160 (7)
F13	0.0732 (10)	0.0366 (7)	0.0566 (9)	−0.0148 (7)	−0.0391 (8)	0.0050 (6)
N1	0.0151 (7)	0.0175 (6)	0.0206 (7)	0.0008 (5)	0.0011 (5)	0.0029 (5)
N2	0.0155 (7)	0.0187 (6)	0.0170 (6)	0.0003 (5)	−0.0012 (5)	−0.0007 (5)
O1	0.0327 (7)	0.0309 (7)	0.0245 (6)	−0.0063 (6)	0.0037 (5)	−0.0049 (5)
O2	0.0326 (7)	0.0279 (6)	0.0211 (6)	−0.0026 (5)	0.0076 (5)	0.0003 (5)
C1	0.0187 (8)	0.0264 (9)	0.0209 (8)	0.0005 (7)	0.0049 (7)	0.0020 (7)
C2	0.0266 (9)	0.0325 (10)	0.0206 (8)	−0.0047 (7)	0.0011 (7)	0.0040 (7)
C3	0.0274 (10)	0.0311 (10)	0.0259 (9)	−0.0083 (8)	0.0015 (7)	−0.0039 (7)
C4	0.0200 (9)	0.0191 (8)	0.0242 (8)	−0.0039 (6)	0.0023 (6)	0.0005 (6)
C5	0.0178 (8)	0.0205 (8)	0.0208 (8)	0.0020 (6)	−0.0018 (6)	0.0030 (6)
C6	0.0170 (8)	0.0214 (8)	0.0182 (8)	0.0016 (6)	−0.0015 (6)	0.0015 (6)
C7	0.0241 (9)	0.0188 (8)	0.0259 (8)	0.0002 (7)	0.0025 (7)	0.0007 (7)
C8	0.0234 (9)	0.0279 (9)	0.0249 (8)	−0.0044 (7)	0.0037 (7)	0.0029 (7)
C9	0.0171 (8)	0.0392 (10)	0.0224 (8)	0.0034 (7)	0.0018 (7)	−0.0016 (8)
C10	0.0278 (9)	0.0290 (9)	0.0228 (9)	0.0075 (7)	−0.0021 (7)	−0.0074 (7)
C11	0.0252 (9)	0.0192 (8)	0.0224 (8)	0.0008 (6)	−0.0026 (7)	0.0005 (7)
C12	0.0254 (9)	0.0194 (8)	0.0247 (8)	0.0052 (7)	−0.0026 (7)	0.0046 (7)
C13	0.0406 (12)	0.0191 (9)	0.0382 (11)	−0.0002 (8)	−0.0001 (9)	0.0063 (8)
C14	0.0238 (9)	0.0201 (8)	0.0180 (7)	0.0033 (7)	−0.0025 (6)	0.0010 (6)
C15	0.0290 (10)	0.0209 (8)	0.0227 (8)	−0.0016 (7)	0.0010 (7)	0.0013 (7)
C16	0.0201 (9)	0.0308 (9)	0.0234 (8)	0.0002 (7)	0.0031 (7)	−0.0026 (7)
C17	0.0180 (8)	0.0205 (8)	0.0208 (8)	0.0024 (6)	−0.0006 (6)	−0.0025 (6)
C18	0.0156 (8)	0.0220 (8)	0.0169 (7)	0.0000 (6)	−0.0009 (6)	−0.0016 (6)
C19	0.0155 (8)	0.0224 (8)	0.0181 (7)	−0.0001 (6)	−0.0023 (6)	−0.0016 (6)
C20	0.0197 (8)	0.0196 (8)	0.0195 (7)	−0.0009 (6)	−0.0023 (6)	−0.0003 (6)
C21	0.0205 (9)	0.0271 (9)	0.0193 (8)	−0.0023 (7)	0.0026 (6)	0.0008 (7)
C22	0.0214 (9)	0.0265 (9)	0.0259 (9)	0.0004 (7)	0.0045 (7)	−0.0056 (7)
C23	0.0294 (10)	0.0184 (8)	0.0339 (9)	−0.0012 (7)	0.0042 (8)	−0.0027 (7)
C24	0.0255 (9)	0.0188 (8)	0.0245 (8)	−0.0036 (7)	0.0043 (7)	0.0020 (7)
C25	0.0170 (8)	0.0227 (8)	0.0223 (8)	−0.0043 (6)	−0.0012 (6)	−0.0010 (6)
C26	0.0289 (10)	0.0228 (8)	0.0234 (9)	−0.0042 (7)	0.0030 (7)	0.0002 (7)

### Geometric parameters (Å, º)

**Table:**

P1—F5	1.5860 (12)	C7—C8	1.391 (2)
P1—F4	1.5953 (12)	C7—H7	0.9500
P1—F2	1.5953 (13)	C8—C9	1.383 (2)
P1—F6	1.5966 (11)	C8—H8	0.9500
P1—F3	1.5977 (12)	C9—C10	1.387 (3)
P1—F1	1.6062 (12)	C9—H9	0.9500
P2—F10	1.578 (2)	C10—C11	1.382 (2)
P2—F7	1.5846 (18)	C10—H10	0.9500
P2—F9	1.5910 (12)	C11—H11	0.9500
P2—F9^i^	1.5910 (12)	C12—C13	1.516 (3)
P2—F8	1.5985 (11)	C12—H12A	0.9900
P2—F8^i^	1.5985 (11)	C12—H12B	0.9900
P3—F13	1.5875 (13)	C13—H13A	0.9800
P3—F13^i^	1.5875 (13)	C13—H13B	0.9800
P3—F12^i^	1.5877 (14)	C13—H13C	0.9800
P3—F12	1.5877 (14)	C14—C15	1.512 (2)
P3—F11^i^	1.6009 (11)	C14—H14A	0.9900
P3—F11	1.6009 (11)	C14—H14B	0.9900
N1—C4	1.514 (2)	C15—H15A	0.9900
N1—C1	1.515 (2)	C15—H15B	0.9900
N1—C12	1.515 (2)	C16—C17	1.509 (2)
N1—C5	1.532 (2)	C16—H16A	0.9900
N2—C14	1.516 (2)	C16—H16B	0.9900
N2—C17	1.516 (2)	C17—H17A	0.9900
N2—C25	1.520 (2)	C17—H17B	0.9900
N2—C18	1.531 (2)	C18—C19	1.511 (2)
O1—C3	1.421 (2)	C18—H18A	0.9900
O1—C2	1.423 (2)	C18—H18B	0.9900
O2—C16	1.421 (2)	C19—C20	1.391 (2)
O2—C15	1.427 (2)	C19—C24	1.393 (2)
C1—C2	1.517 (2)	C20—C21	1.388 (2)
C1—H1A	0.9900	C20—H20	0.9500
C1—H1B	0.9900	C21—C22	1.389 (2)
C2—H2A	0.9900	C21—H21	0.9500
C2—H2B	0.9900	C22—C23	1.384 (3)
C3—C4	1.515 (2)	C22—H22	0.9500
C3—H3A	0.9900	C23—C24	1.384 (3)
C3—H3B	0.9900	C23—H23	0.9500
C4—H4A	0.9900	C24—H24	0.9500
C4—H4B	0.9900	C25—C26	1.513 (2)
C5—C6	1.501 (2)	C25—H25A	0.9900
C5—H5A	0.9900	C25—H25B	0.9900
C5—H5B	0.9900	C26—H26A	0.9800
C6—C11	1.390 (2)	C26—H26B	0.9800
C6—C7	1.396 (2)	C26—H26C	0.9800
			
F5—P1—F4	90.08 (8)	C11—C6—C5	121.85 (15)
F5—P1—F2	91.00 (8)	C7—C6—C5	119.14 (15)
F4—P1—F2	178.87 (8)	C8—C7—C6	120.26 (16)
F5—P1—F6	90.47 (6)	C8—C7—H7	119.9
F4—P1—F6	89.80 (6)	C6—C7—H7	119.9
F2—P1—F6	90.54 (6)	C9—C8—C7	120.09 (16)
F5—P1—F3	179.12 (8)	C9—C8—H8	120.0
F4—P1—F3	89.58 (8)	C7—C8—H8	120.0
F2—P1—F3	89.34 (8)	C8—C9—C10	119.92 (16)
F6—P1—F3	90.34 (6)	C8—C9—H9	120.0
F5—P1—F1	89.54 (6)	C10—C9—H9	120.0
F4—P1—F1	90.32 (7)	C11—C10—C9	120.04 (16)
F2—P1—F1	89.33 (7)	C11—C10—H10	120.0
F6—P1—F1	179.88 (9)	C9—C10—H10	120.0
F3—P1—F1	89.65 (6)	C10—C11—C6	120.73 (16)
F10—P2—F7	180.0	C10—C11—H11	119.6
F10—P2—F9	90.71 (7)	C6—C11—H11	119.6
F7—P2—F9	89.29 (7)	N1—C12—C13	114.87 (15)
F10—P2—F9^i^	90.72 (7)	N1—C12—H12A	108.6
F7—P2—F9^i^	89.29 (7)	C13—C12—H12A	108.6
F9—P2—F9^i^	178.57 (13)	N1—C12—H12B	108.6
F10—P2—F8	89.67 (6)	C13—C12—H12B	108.6
F7—P2—F8	90.33 (6)	H12A—C12—H12B	107.5
F9—P2—F8	90.61 (6)	C12—C13—H13A	109.5
F9^i^—P2—F8	89.40 (6)	C12—C13—H13B	109.5
F10—P2—F8^i^	89.67 (6)	H13A—C13—H13B	109.5
F7—P2—F8^i^	90.33 (6)	C12—C13—H13C	109.5
F9—P2—F8^i^	89.40 (6)	H13A—C13—H13C	109.5
F9^i^—P2—F8^i^	90.61 (6)	H13B—C13—H13C	109.5
F8—P2—F8^i^	179.33 (11)	C15—C14—N2	112.56 (14)
F13—P3—F13^i^	90.13 (13)	C15—C14—H14A	109.1
F13—P3—F12^i^	179.46 (9)	N2—C14—H14A	109.1
F13^i^—P3—F12^i^	89.60 (8)	C15—C14—H14B	109.1
F13—P3—F12	89.60 (8)	N2—C14—H14B	109.1
F13^i^—P3—F12	179.46 (9)	H14A—C14—H14B	107.8
F12^i^—P3—F12	90.67 (13)	O2—C15—C14	111.26 (14)
F13—P3—F11^i^	90.49 (7)	O2—C15—H15A	109.4
F13^i^—P3—F11^i^	89.41 (7)	C14—C15—H15A	109.4
F12^i^—P3—F11^i^	89.97 (7)	O2—C15—H15B	109.4
F12—P3—F11^i^	90.12 (7)	C14—C15—H15B	109.4
F13—P3—F11	89.41 (7)	H15A—C15—H15B	108.0
F13^i^—P3—F11	90.49 (7)	O2—C16—C17	111.80 (15)
F12^i^—P3—F11	90.12 (7)	O2—C16—H16A	109.3
F12—P3—F11	89.97 (7)	C17—C16—H16A	109.3
F11^i^—P3—F11	179.86 (12)	O2—C16—H16B	109.3
C4—N1—C1	107.14 (12)	C17—C16—H16B	109.3
C4—N1—C12	109.23 (13)	H16A—C16—H16B	107.9
C1—N1—C12	113.09 (13)	C16—C17—N2	112.34 (14)
C4—N1—C5	109.47 (12)	C16—C17—H17A	109.1
C1—N1—C5	107.19 (12)	N2—C17—H17A	109.1
C12—N1—C5	110.62 (13)	C16—C17—H17B	109.1
C14—N2—C17	107.49 (12)	N2—C17—H17B	109.1
C14—N2—C25	107.22 (12)	H17A—C17—H17B	107.9
C17—N2—C25	109.71 (12)	C19—C18—N2	114.29 (13)
C14—N2—C18	111.97 (12)	C19—C18—H18A	108.7
C17—N2—C18	109.86 (12)	N2—C18—H18A	108.7
C25—N2—C18	110.49 (12)	C19—C18—H18B	108.7
C3—O1—C2	109.58 (13)	N2—C18—H18B	108.7
C16—O2—C15	109.60 (13)	H18A—C18—H18B	107.6
N1—C1—C2	111.93 (14)	C20—C19—C24	118.82 (15)
N1—C1—H1A	109.2	C20—C19—C18	120.37 (15)
C2—C1—H1A	109.2	C24—C19—C18	120.71 (15)
N1—C1—H1B	109.2	C21—C20—C19	120.50 (16)
C2—C1—H1B	109.2	C21—C20—H20	119.7
H1A—C1—H1B	107.9	C19—C20—H20	119.7
O1—C2—C1	110.46 (14)	C20—C21—C22	120.23 (16)
O1—C2—H2A	109.6	C20—C21—H21	119.9
C1—C2—H2A	109.6	C22—C21—H21	119.9
O1—C2—H2B	109.6	C23—C22—C21	119.48 (16)
C1—C2—H2B	109.6	C23—C22—H22	120.3
H2A—C2—H2B	108.1	C21—C22—H22	120.3
O1—C3—C4	110.93 (15)	C22—C23—C24	120.36 (16)
O1—C3—H3A	109.5	C22—C23—H23	119.8
C4—C3—H3A	109.5	C24—C23—H23	119.8
O1—C3—H3B	109.5	C23—C24—C19	120.60 (16)
C4—C3—H3B	109.5	C23—C24—H24	119.7
H3A—C3—H3B	108.0	C19—C24—H24	119.7
N1—C4—C3	112.16 (14)	C26—C25—N2	115.19 (14)
N1—C4—H4A	109.2	C26—C25—H25A	108.5
C3—C4—H4A	109.2	N2—C25—H25A	108.5
N1—C4—H4B	109.2	C26—C25—H25B	108.5
C3—C4—H4B	109.2	N2—C25—H25B	108.5
H4A—C4—H4B	107.9	H25A—C25—H25B	107.5
C6—C5—N1	114.64 (13)	C25—C26—H26A	109.5
C6—C5—H5A	108.6	C25—C26—H26B	109.5
N1—C5—H5A	108.6	H26A—C26—H26B	109.5
C6—C5—H5B	108.6	C25—C26—H26C	109.5
N1—C5—H5B	108.6	H26A—C26—H26C	109.5
H5A—C5—H5B	107.6	H26B—C26—H26C	109.5
C11—C6—C7	118.93 (15)		
			
C4—N1—C1—C2	−52.21 (17)	C17—N2—C14—C15	−50.76 (17)
C12—N1—C1—C2	68.20 (18)	C25—N2—C14—C15	−168.65 (13)
C5—N1—C1—C2	−169.62 (13)	C18—N2—C14—C15	69.99 (17)
C3—O1—C2—C1	−62.46 (19)	C16—O2—C15—C14	−60.77 (18)
N1—C1—C2—O1	59.13 (19)	N2—C14—C15—O2	57.50 (18)
C2—O1—C3—C4	62.01 (19)	C15—O2—C16—C17	60.96 (18)
C1—N1—C4—C3	51.63 (18)	O2—C16—C17—N2	−57.46 (18)
C12—N1—C4—C3	−71.20 (17)	C14—N2—C17—C16	50.49 (17)
C5—N1—C4—C3	167.54 (14)	C25—N2—C17—C16	166.76 (13)
O1—C3—C4—N1	−58.05 (19)	C18—N2—C17—C16	−71.58 (17)
C4—N1—C5—C6	60.64 (17)	C14—N2—C18—C19	63.20 (17)
C1—N1—C5—C6	176.52 (13)	C17—N2—C18—C19	−177.44 (14)
C12—N1—C5—C6	−59.77 (17)	C25—N2—C18—C19	−56.25 (17)
N1—C5—C6—C11	92.12 (18)	N2—C18—C19—C20	93.27 (18)
N1—C5—C6—C7	−91.19 (18)	N2—C18—C19—C24	−90.25 (19)
C11—C6—C7—C8	−0.7 (3)	C24—C19—C20—C21	0.7 (2)
C5—C6—C7—C8	−177.49 (16)	C18—C19—C20—C21	177.26 (15)
C6—C7—C8—C9	−0.9 (3)	C19—C20—C21—C22	0.3 (3)
C7—C8—C9—C10	1.4 (3)	C20—C21—C22—C23	−0.6 (3)
C8—C9—C10—C11	−0.4 (3)	C21—C22—C23—C24	−0.1 (3)
C9—C10—C11—C6	−1.2 (3)	C22—C23—C24—C19	1.1 (3)
C7—C6—C11—C10	1.7 (3)	C20—C19—C24—C23	−1.4 (3)
C5—C6—C11—C10	178.43 (16)	C18—C19—C24—C23	−177.96 (17)
C4—N1—C12—C13	174.38 (15)	C14—N2—C25—C26	170.87 (14)
C1—N1—C12—C13	55.17 (19)	C17—N2—C25—C26	54.43 (18)
C5—N1—C12—C13	−65.07 (19)	C18—N2—C25—C26	−66.85 (17)

Symmetry code: (i) −*x*, −*y*, *z*.

### Hydrogen-bond geometry (Å, º)

**Table:**

*D*—H···*A*	*D*—H	H···*A*	*D*···*A*	*D*—H···*A*
C1—H1*A*···F4	0.99	2.41	3.211 (2)	138
C3—H3*B*···F2^ii^	0.99	2.47	3.389 (2)	155
C5—H5*B*···F11^iii^	0.99	2.48	3.365 (2)	150
C10—H10···F10^iv^	0.95	2.50	3.288 (2)	141
C17—H17*A*···F8^v^	0.99	2.37	3.329 (2)	162
C17—H17*B*···F1	0.99	2.46	3.357 (2)	150
C25—H25*B*···F1	0.99	2.53	3.388 (2)	145

Symmetry codes: (ii) −*x*+3/4, *y*+1/4, *z*−1/4; (iii) *x*+1/4, −*y*+1/4, *z*+1/4; (iv) −*x*+1/2, −*y*, *z*−1/2; (v) −*x*+1/2, −*y*, *z*+1/2.
